# Progesterone signalling in broiler skeletal muscle is associated with divergent feed efficiency

**DOI:** 10.1186/s12918-017-0396-2

**Published:** 2017-02-24

**Authors:** Walter Bottje, Byung-Whi Kong, Antonio Reverter, Ashley J. Waardenberg, Kentu Lassiter, Nicholas J. Hudson

**Affiliations:** 1 Department of Poultry Science, University of Arkansas, Fayetteville, AR USA; 2 Agriculture, Commonwealth Science and Industrial Research Organisation, 306 Carmody Road, Brisbane, QLD 4072 Australia; 3 Children’s Medical Research Institute, University of Sydney, 214 Hawkesbury Road, Westmead, NSW 2145 Australia; 4 School of Agriculture and Food Science, University of Queensland, Gatton, QLD 4343 Australia

**Keywords:** Feed efficiency, Mitochondria, Progesterone

## Abstract

**Background:**

We contrast the *pectoralis* muscle transcriptomes of broilers selected from within a single genetic line expressing divergent feed efficiency (FE) in an effort to improve our understanding of the mechanistic basis of FE.

**Results:**

Application of a virtual muscle model to gene expression data pointed to a coordinated reduction in slow twitch muscle isoforms of the contractile apparatus (*MYH15*, *TPM3*, *MYOZ2*, *TNNI1*, *MYL2*, *MYOM3*, *CSRP3*, *TNNT2*), consistent with diminishment in associated slow machinery (myoglobin and phospholamban) in the high FE animals. These data are in line with the repeated transition from red slow to white fast muscle fibres observed in agricultural species selected on mass and FE. Surprisingly, we found that the expression of 699 genes encoding the broiler mitoproteome is modestly–but significantly–biased towards the high FE group, suggesting a slightly elevated mitochondrial content. This is contrary to expectation based on the slow muscle isoform data and theoretical physiological capacity arguments. Reassuringly, the extreme 40 most DE genes can successfully cluster the 12 individuals into the appropriate FE treatment group. Functional groups contained in this DE gene list include metabolic proteins (including opposing patterns of *CA3* and *CA4*), mitochondrial proteins (*CKMT1A*), oxidative status (*SEPP1*, *HIG2A*) and cholesterol homeostasis (*APOA1*, *INSIG1*). We applied a differential network method (Regulatory Impact Factors) whose aim is to use patterns of differential co-expression to detect regulatory molecules transcriptionally rewired between the groups. This analysis clearly points to alterations in progesterone signalling (via the receptor *PGR*) as the major driver. We show the progesterone receptor localises to the mitochondria in a quail muscle cell line.

**Conclusions:**

Progesterone is sometimes used in the cattle industry in exogenous hormone mixes that lead to a ~20% increase in FE. Because the progesterone receptor can localise to avian mitochondria, our data continue to point to muscle mitochondrial metabolism as an important component of the phenotypic expression of variation in broiler FE.

**Electronic supplementary material:**

The online version of this article (doi:10.1186/s12918-017-0396-2) contains supplementary material, which is available to authorized users.

## Background

In a resource-constrained world supporting a rapidly growing human population there is great interest in enhancing the production efficiency of our major animal and plant food industries. Feed is the single largest commercial cost in animal production [1]. Any inefficiency not only affects the bottom line but also negatively impacts resource usage (e.g. water and energy) and waste production (e.g. urine). Consequently, there is considerable economic and environmental interest in increasing animal feed conversion efficiency. Small increases in feed efficiency across numerous animals can have a large industrial and environmental impact.

Broiler chickens are the most feed efficient of all the vertebrates, with some companies boasting a conversion of just ~1.5 units dry weight intake per unit wet weight gain [2]. This makes broilers particularly valuable as biological models for understanding the mechanistic basis of feed efficiency. Along with other avian meat producing species, *cf*. turkeys, pheasants, partridge, grouse and quails, chickens are members of the Phasianidae taxonomic clade, the largest branch of the Galliformes. The members of this group tend to be sedentary, resident ground-dwelling birds that use short, burst flights to escape predators [3, 4]. This behaviour explains the functionally unusual breast muscle metabolism of the ancestral birds such as the Red Junglefowl (*Gallus gallus*) progenitor of modern domestic chickens, which is dominated by explosive, fast twitch contractile isoforms and a relatively low oxidative capacity metabolism.

The birds we chose for this analysis are pedigree broiler males (PedM) from a population of highly inbred, commercial animals subject to very strong historical selection for high muscle mass and elevated feed efficiency (FE) [5]. The significant difference in FE (g gain/g feed) between the two treatment groups is in the order of 1.4 fold in absolute terms. Some of the variation in the FE phenotype presumably derives from biological modifications in the non-muscle tissues. This logic, coupled with the prior application of microarray technology on the same individuals [5], gave us the expectation that any molecular differences we may detect in the breast muscle between the two groups would be fairly subtle. Nevertheless, it has been established for some time that commercial broilers possess a muscle structure that is, histologically speaking, composed almost exclusively of low mitochondrial content type IIB glycolytic fibres [3]. This fibre homogeneity is advantageous as it provides a very stable transcriptional background against which to perform the various molecular analyses.

Here, we have used Illumina RNA sequencing technology to screen the breast muscle transcriptomes of the high FE (HFE) and low FE (LFE) groups. We aimed to both test existing hypotheses as well as generate new ones. We have previously argued for a role of both isolated mitochondrial physiology [5–20] and overall tissue mitochondrial content [21] in driving variation in production animal feed efficiency. Therefore, as part of our analyses we have specifically harvested the data for genes encoding mitochondrial proteins, finding expression was skewed towards the HFE birds. Furthermore, we have also used a combination of basic differential expression (DE) and a recently developed differential connectivity (DC) algorithm called Regulatory Impact Factor (RIF) analysis to screen the data in an unbiased fashion [22–24]. The RIF algorithm detects regulatory molecules that are transcriptionally rewired between the two states, irrespective of whether that molecule is DE itself. This is a potent approach for the identification of Transcription Factors that tend to be lowly and stably expressed at the mRNA level [25], but whose functional activity is controlled at the protein level through changes in cellular localisation, ligand binding or post-translational modifications. This analysis points to progesterone signalling through the progesterone receptor as a key molecular driver.

## Methods

### Animal resources and phenotypes

We used breast skeletal muscle samples from 12 male broilers divergent in feed efficiency as previously described [5]. Male broilers were raised under standard industry practices and euthanized using carbon dioxide asphyxiation. Cobb-Vantress Inc. adheres to internal industrial standard guidelines for animal welfare and has standard operating procedures for euthanasia, dissection and other experimental techniques. In brief, the 12 broilers were derived from a single genetic line of a commercial Cobb-Vantress Inc. population. Both the HFE (*n* = 6) and LFE (*n* = 6) birds were sub-sampled from the extremes of an original group of 100 birds. In turn, those 100 birds were selected as the most efficient from a larger population of 300 birds. The high FE group had greater body weight gain from 6 to 7 weeks but consumed the same amount of feed as the LFE group. For the purposes of this manuscript we have defined FE as the gain in bird mass relative to feed intake, such that a higher number equates to a more efficient bird. In our case, the HFE birds yielded efficiencies of 0.65 ± 0.01 compared to 0.46 ± 0.01 (g gain to g feed intake) for the LFE birds. This animal resource is noteworthy in the sense that the two groups are not divergently selected. This is an important distinction compared to other research populations in this area. For example, the observed DE in divergent selection experiments could be a consequence of functionally irrelevant founder effects that have accumulated over subsequent matings. In our case the birds are from the same line and generation, but separated on the basis of 1 week of FCR testing in a controlled experimental environment.

### Mapping, counting and normalising the mRNA reads

After phenol chloroform RNA extraction we submitted our samples to the Research Support Facility at Michigan State University (East Lansing, MI) for Illumina HiSeq 100 base pair paired end read sequencing. In brief, we used CLC Genomics Workbench 8 to map the reads to *Gallus gallus* genome assembly version 4. The CLC software follows the analytical pipeline recommended in [26]. We log2 transformed the RPM data to stabilise the variance and then performed a further quantile normalisation. The impact of the two levels of normalisation on whether the 12 individual samples could discriminate into the treatment groups of origin was confirmed by Principal Component Analysis (Additional file 1).

### Differential expression

**Fig. 1:**
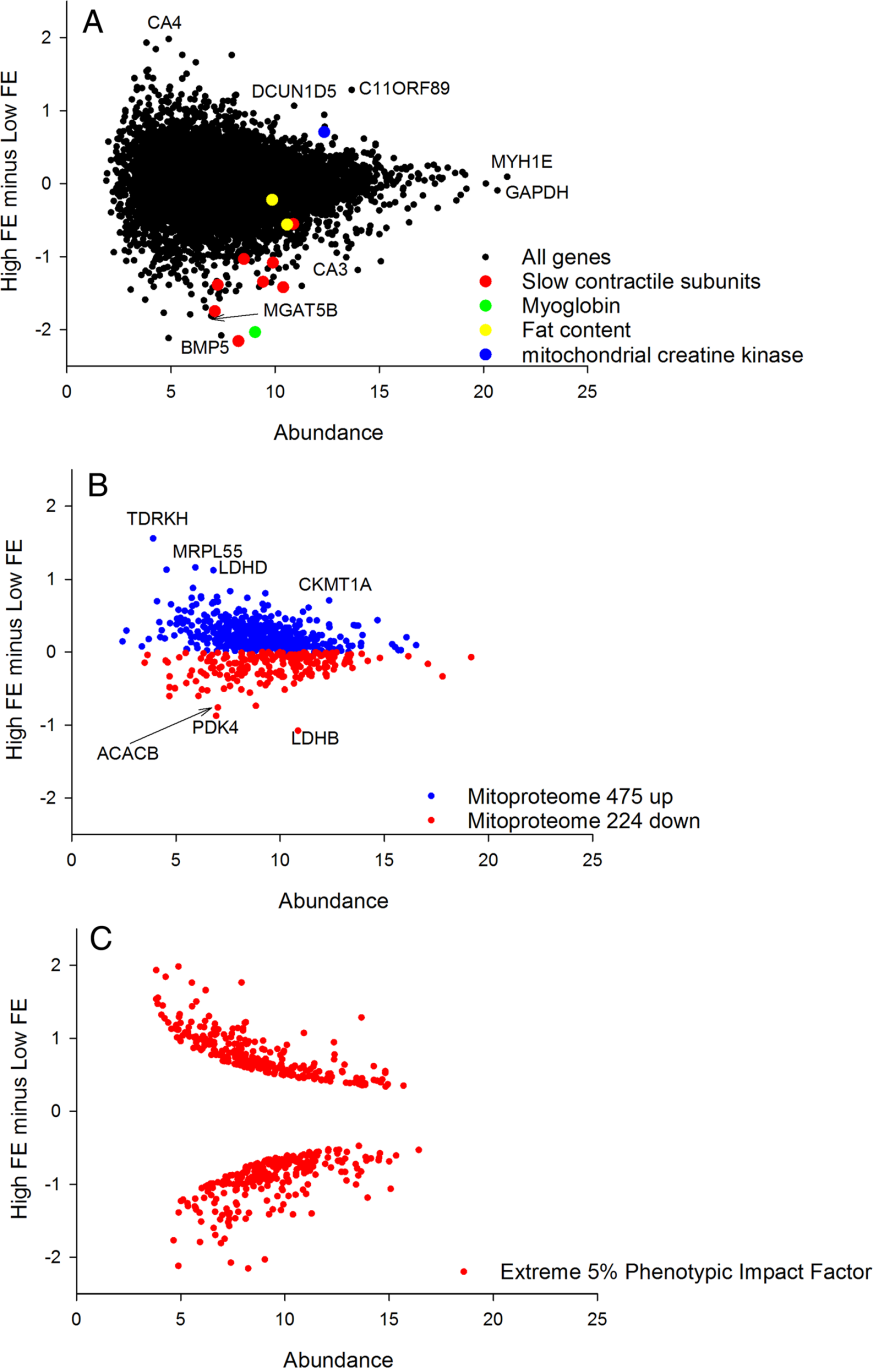
**a** The slow contractile subunits highlighted are *MYH15, TPM3, MYOZ2, TNNI1, MYL2, TNNT2* and *CSRP3* consistent with a shift in fibre composition towards the fast glycolytic and away from the slow aerobic fibres in HFE birds. **b** The mRNA encoding the mitoproteome is significantly skewed towards the HFE consistent with a slight increase in mitochondrial content. **c** The extreme 5% PIF used as input for the RIF analysis have been highlighted. These DE genes are distributed evenly across the MA plot, closely tracking the overall shape of the plot

To compare the 2 treatment groups we plotted the MA (i.e. Minus Average) plot comparing HFE birds to LFE birds (Fig. 1 panel a). The x axis, A, represents the average abundance in the 2 groups, and the y axis, M, is HFE average transcript abundance minus LFE average transcript abundance. A subset of functionally relevant outlier DE genes was chosen for annotation on the plot.

### mRNA encoding the mitoproteome

We downloaded the complete human mitoproteome from http://mitominer.mrc-mbu.cam.ac.uk/release-3.1/begin.do generating a list of 1046 nuclear and mitochondrial genes encoding mitochondrial proteins. We converted the protein names to gene names, and found 699 matches in our chicken RNAseq data. The mitoproteome was then plotted in MA format, and all those with higher values in the HFE group were colour coded blue and those with lower values in the HFE colour coded red (Fig. 1 panel b). The skew in distribution away from the null expectation of 50:50 was quantified by binomial statistics. This approach formalises the extent to which the mitoproteome data is biased to one or the other of the two groups.

### Phenotypic Impact Factor (PIF)

We next computed a modified DE called PIF (DE multiplied by abundance) which we have found to have a number of appealing characteristics, both numerical and biological. From a numerical perspective it can be used to establish extreme DE genes in a way that accounts for the structure of the distribution of the data (Fig. 1 panel c). It de-emphasises lowly abundant genes that are inherently noisy as they approach the detection limit of the technology. It also draws together the 2 major sources of variation in gene expression data into a single metric which can be used for comparison purposes. From a biological perspective PIF analysis is useful as it gives more meaningful functional enrichments than ranking on DE, including better characterising muscle fibre composition shifts in muscle transcriptome data [23]. Also, PIF is a foundation of the RIF differential network analysis (below) where the target DE molecules are first defined by extreme PIF, and then the regulators differential co-expression to those targets is integrated into a differential connectivity metric. We ranked on PIF and assessed functional enrichment at the extremes of the list using GOrilla [27].

### VMus3D Muscle Model

**Fig. 2:**
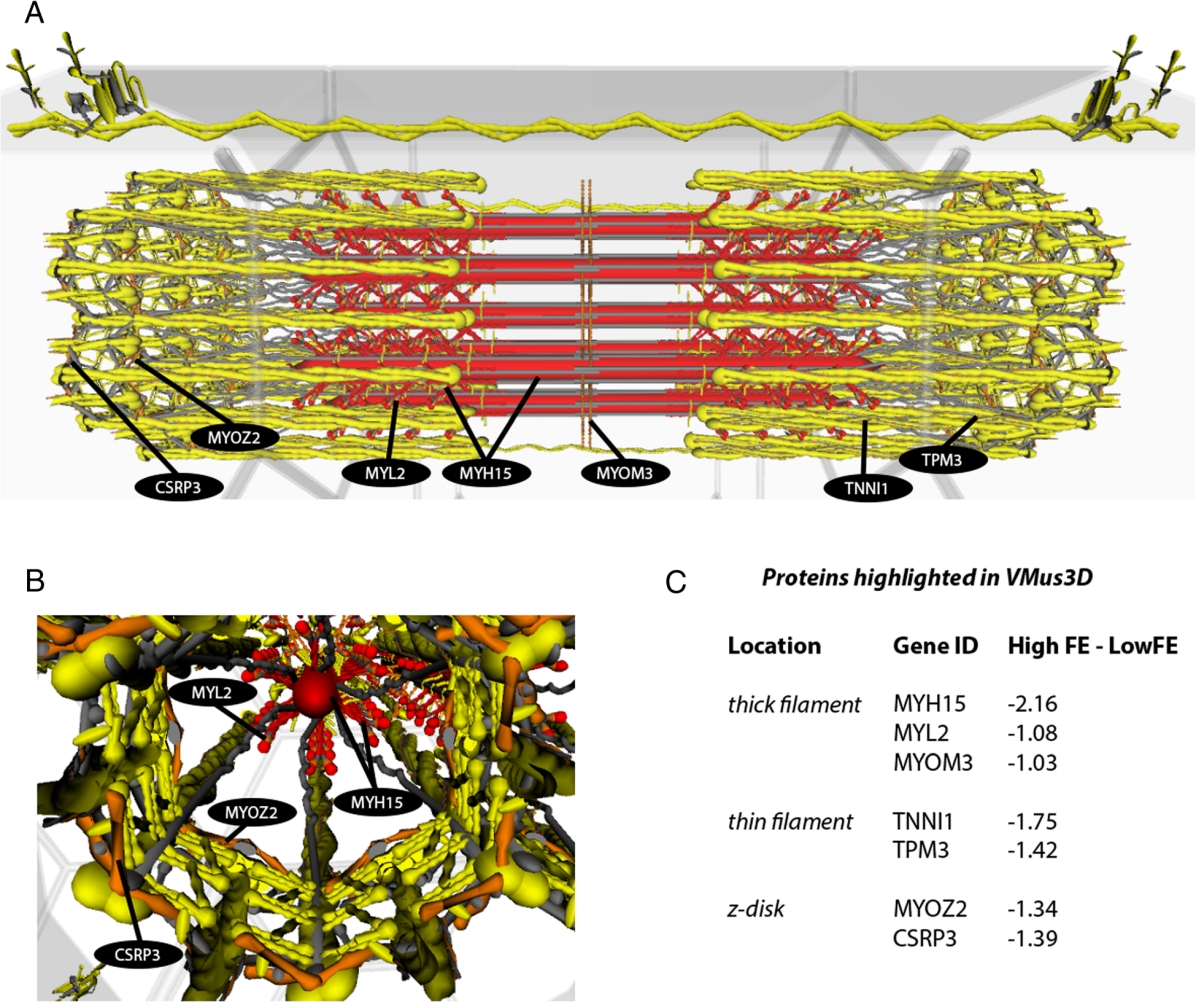
3D muscle model visualisation illustrating the downregulation of slow subunits in HFE birds consistent with a whiter, sprint muscle phenotype. **a**. Longitudinal view of the contractile apparatus with coordinated DE changes of slow unit proteins highlighted. **b**. Z-disk proteins differentially expressed. **c**. Protein locations across the contractile apparatus and their corresponding values. Colours correspond to the level of DE (M-value) as follows: blue (≥ 2), green (< 2 and ≥ 1), yellow (> −1 and < 1), orange (≤ −1 and > −2) and red (≤ −2)

To visualise spatial locations of proteins encoded by DE genes derived from the HFE versus LFE birds we utilised the Virtual Muscle 3D (VMus3D). VMus3D, described in detail here [28, 29], is an annotated 3-dimensional representation of key contractile protein complexes, including thick, thin, z-disc and costameric complexes, and their relative protein spatial locations. VMus3D is a database driven visualisation tool that enables mapping of gene expression changes onto their encoded proteins, represented as an extensible 3D object, via the Document Object Model. Here, we mapped M-values onto the VMus3D and coloured change as follows: blue (≥2), green (< 2 and ≥ 1), yellow (> −1 and < 1), orange (≤ −1 and > −2) and red (≤ −2) (Fig. 2).

### Permut Matrix clustering on extreme PIF

**Fig. 3:**
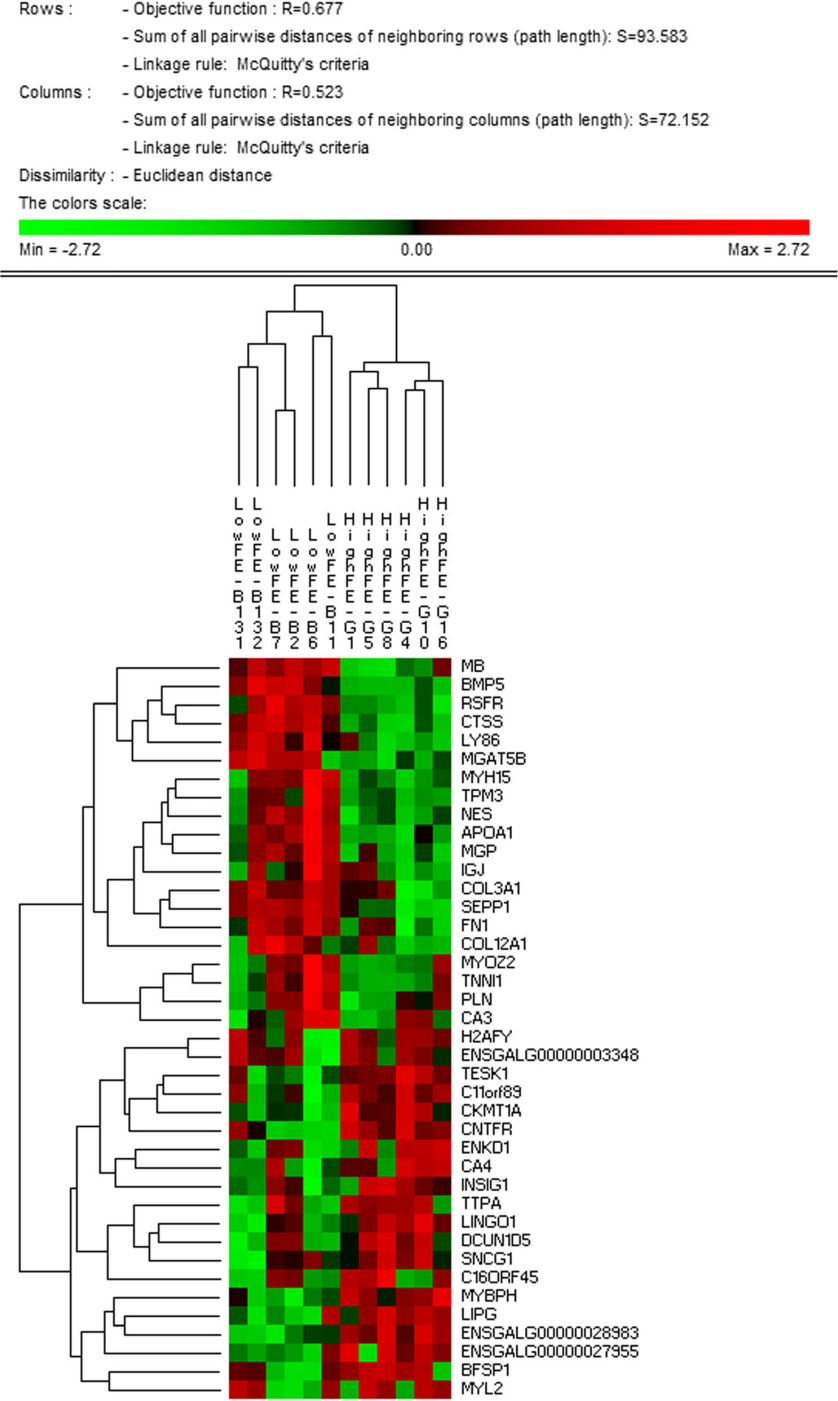
A hierarchically clustered heatmap showing the expression patterns of the 40 most extreme PIF mRNA. This panel of 40 genes which includes mRNA encoding muscle structural proteins and mitochondrial proteins can correctly discriminate the two treatment groups

The top 20 up and top 20 downregulated genes (by PIF) were identified as described above. A matrix was formed comprising as many rows as genes (40) and as many columns as birds (12) with each cell containing the normalised gene expression. This matrix was imported into PermutMatrix software [30] for hierarchical clustering (Fig. 3).

### Regulatory Impact Factor (RIF) analysis

The purpose of RIF is to identify regulatory molecules whose activity can change independent of any change in gene expression level. This includes Transcription Factors whose activity may be influenced by ligand binding, co-factor binding, cellular localisation and other post-translational processes. The method works by establishing different patterns of network connectivity in the two states (here HFE versus LFE). RIF1 and RIF2 are two versions of essentially the same analysis. In both cases the abundance and differential expression of the ‘target genes’ is exploited in conjunction with the differential co-expression of the ‘regulators’ to those ‘targets.’ The output of both versions has been plotted and reported here.

**Fig. 4:**
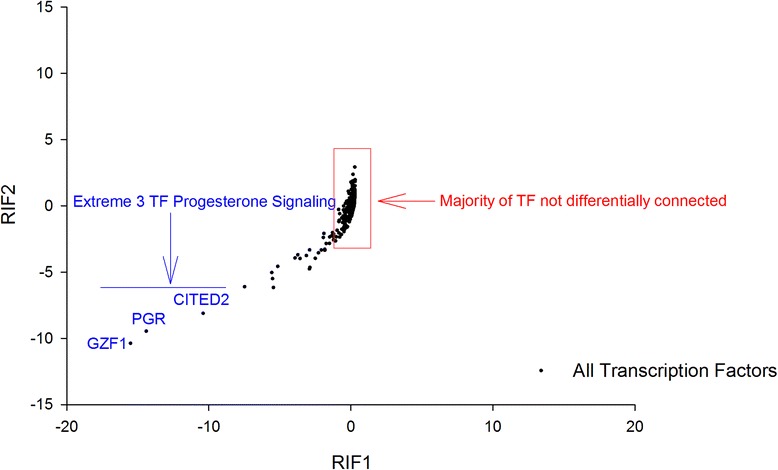
The extremely differentially connected TF as illustrated by RIF1 and RIF2 scores. Progesterone receptor and 2 other TF involved in progesterone signalling are awarded extreme scores by both metrics

We computed RIF1 and RIF2 as previously described [22–24]. This procedure exploits global patterns of differential co-expression (or ‘differential wiring’) to infer those regulatory molecules whose behaviour is systematically different in a contrast of interest, in this case the HFE versus LFE birds. Herein, the experimental contrast was HFE vs. LFE and the RIF metrics for the *r*-th regulator (*r* = 1, 2, …898) were computed using the following formulae:

$$ \mathrm{R}\mathrm{I}\mathrm{F}{1}_r=\frac{1}{{\mathrm{n}}_{\mathrm{DE}}}{\displaystyle \sum_{j=1}^{j={\mathrm{n}}_{\mathrm{DE}}}{x}_j\times {d}_j\times {\mathrm{DC}}_{rj}^2} $$

and

$$ \mathrm{R}\mathrm{I}\mathrm{F}{2}_r=\frac{1}{{\mathrm{n}}_{\mathrm{DE}}}{\displaystyle \sum_{j=1}^{j={\mathrm{n}}_{\mathrm{DE}}}\left[{\left({x}_j^{\mathrm{HFE}}\times {r}_{rj}^{\mathrm{HFE}}\right)}^2-{\left({x}_j^{\mathrm{LFE}}\times {r}_{rj}^{\mathrm{LFE}}\right)}^2\right]} $$

where n_DE_ represented the number of DE genes; *x*_*j*_ was the average expression of the *j*-th DE gene across all time points; *d*_*j*_ was the DE of the *j*-th gene in the HFE vs. LFE contrast; DC_*rj*_ was the differential co-expression between the *r*-th regulator and the *j*-th DE gene, and computed from the difference between *r*_*rj*_^HFE^ and *r*_*rj*_^LFE^, the correlation co-expression between the *r*-th regulator and the *j*-th DE gene in the HFE and LFE samples, respectively; finally, *x*_*j*_^HFE^ and *x*_*j*_^LFE^ represented the average expression of the j-th DE or TS gene in the HFE and LFE samples, respectively. The RIF1 and RIF2 output were simultaneously plotted (Fig. 4).

### Quantitative PCR technical validation

To independently validate our RNAseq normalisation method and method to detect DE, we selected a subset of nine representative genes for qPCR amplification. These were mRNA encoding slow twitch subunits (*MYH15*, *TPM3*, *MYOZ2*, *TNNI1*, *MYBPC1*), slow muscle associated proteins (*MB*, *CA3*) and muscle fat content (*PLN* and *FABP4*). We used PCR cycling conditions as previously described [5, 11]. Primers are detailed in Additional file 2.

In brief, one microgram of total RNA was obtained from 6 muscle samples each for high and low FE pedigree broilers for general validation of the RNAseq results and for specific confirmation. RNA was converted into cDNA with qScriptTM cDNA SuperMix (Quanta Biosciences, Gaithersburg, MD) following the manufacturer’s instructions. The cDNA samples were diluted in a 1:10 ratio and a portion (2 μL) of the cDNA was subjected to a real-time quantitative PCR (qPCR) reaction using an ABI prism 7500HT system (ThermoFisher Scientific, Waltham, MA) with PowerUpTM SYBR® Green Master Mix (ThermoFisher Scientific, Waltham, MA). The specific oligonucleotide primers were designed by the PRIMER3 program (http://frodo.wi.mit.edu). The conditions of real-time qPCR amplification were 1 cycle at 95 °C for 2 min, 40 cycles at 95 °C for 30 s, 65 °C for 30 s. The chicken glyceraldehyde 3-phosphate dehydrogenase (GAPDH) gene was used as the internal control. Dissociation curves were performed at the end of amplification for validating data quality. All qPCR reactions were performed 3 times and the values of average cycle threshold (Ct) were determined for each sample, and 2 − ΔΔCt values for the comparison of HFE and LFE muscles were used for relative quantification by fold-change and statistical significance.

### Quail muscle (QM7) cell culture and treatments

QM7 cells were grown in M199 medium (Life Technologies, Grand Island, NY) with 10% fetal bovine serum (Life Technologies), 10% tryptose phosphate (Sigma-Aldrich, St. Louis, MO), and 1% penicillin-streptomycin (Biobasic, Amherst, NY) at 37 °C under a humidified atmosphere of 5% CO2 and 95% air. At 80–90% confluence, cells were synchronized overnight in serum-free medium.

### Progesterone receptor immunofluorescence

Immunofluorescence was performed as previously described [31] with modifications. Briefly, cells were grown to 50–60% confluence in chamber slides (Lab-Tek, Hatfield, PA) followed by methanol fixation for 10 min at 20 °C. Serum free blocking buffer (Dako, Carpinteria, CA), and the cells incubated with rabbit anti-progesterone receptor antibody overnight at 4 °C. Cells were treated with MitoTracker Deep Red (Molecular Probes, Inc., Eugene OR) and visualized with Alexa Fluor 488-conjugated secondary antibody (Molecular Probes, Life Technologies). After DAPI counterstaining, a cover slip was placed on slides with Vectashield (Vector Laboratories, Burlingame, CA). Images were obtained using the Zeiss Imager M2 with a 20X Plan-APOCHROMAT 20X/0.8 objective and a 100X EC PLANNEOFLUOR 100X/1.3 oil objective. The Alexa Fluor 488 fluorophore was observed through filter set 38 1031-346 with an excitation of BP 470/40, beamsplitter of FT 495, and emission spectrum of BP 525/50. Differential interference contrast images were collected using DIC M27 condensers. The Alexa Fluor 488 fluorophore was excited for 500 ms prior to capturing each image using an Axio Cam MR3 camera. All analysis was performed using AxioVision SE64 4.9.1 SP1 software (Carl Zeiss Microscopy 2006–2013).

### Statistical analysis

Given the two treatment groups are so similar and that multiple testing penalties can be very severe in this kind of experimental design we elected not to identify significantly DE genes on a gene-by-gene basis. Rather, we used a range of systems-wide analyses to interpret our data. This approach has the advantage of not being reliant on the performance of any given gene, but rather draws on information present in entire functional groups of genes. For basic functional enrichment analysis we used GOrilla [27]. Here, we examined functional enrichment in the extreme 260 up and downregulated genes (nominal 5% or 520 out of the total 10, 416) genes ranked by PIF, compared to a background list consisting of all genome-wide data; both *P* and FDR *q*-values reported. This nominal 5% DE genes also represent the target DE genes for the RIF analysis. For the mitoproteome analysis we used binomial statistics to quantify the skew for all mRNA encoding mitochondrial proteins. For the RIF analysis we report on extreme outlier regulators only.

## Results

Twelve RNAseq libraries were constructed using breast muscle RNA samples from 6 HFE and 6 LFE broilers. In total, 804 million 100 bp sequences were obtained with an average of 67 million reads per sample and 80% of the reads were mapped to the *G. gallus* reference genome assembly. After mapping, counting and normalising we returned data for 12,814 genes. We screened for those mRNA with no missing values across the 12 samples. This gave a total 10,412 genes which were used for all subsequent analysis. Both RPM and RPKM values were computed and compared by correlation analysis (Additional file 3). To achieve this we calculated the PIF for each gene (abundance multiplied by DE) and correlated the PIF scores by the two methods. Given the high correlation (0.93) and the concern that the additional normalisation used in generating RPKM values can theoretically introduce a variety of problems [32], we elected to proceed with the normalised RPM values.

### Principal component analysis

The raw read count data could not discriminate the birds into treatment group of origin. Log2 stabilising the data allowed separation of individuals into the two groups with one exception. This separation by treatment group was reinforced by a subsequent quantile normalisation which standardises the distributions of the reads for each sample (Additional file 1). This separation provided a quality check that our normalisation approach had maintained the treatment differences without introducing any systematic bias. Further validation that the normalisation approach did not introduce bias was provided by determination that the MA plot was centred on 0.

### Differential expression between HFE and LFE birds

We used a modified DE, named PIF for: 1) functional enrichment analysis in GOrilla, 2) to generate a panel of genes that can discriminate the birds into treatment group (below) and, 3) as the prelude step identifying the targets for the RIF analysis. PIF is computed by multiplying DE by abundance i.e. the two sources of variation in expression data. The top enrichment among the 260 upregulated genes (top 2.5% out of 10,412) in HFE by PIF is “cholesterol biosynthesis” (*P* = 0.00027; FDR *q* value = 0.1) based on the presence of *DHCR24*, *INSIG1*, *FDPS*, *SC5D* and *MVD*. The top enrichment among the 260 downregulated genes in HFE by PIF is “extracellular matrix organisation” (*P* = 1.03 E-26; FDR *q* value = 1.24 E-22) based on the presence of *FMOD, ITGA4, ACTN1, SMOC2, FN1, ITGA8, MFAP5, CMA1, TNC, GSN, TLL1, NRXN1, COL1A2, COL3A1, CCDC80, DCN, LTBP3, CYP1B1, ITGB2, NCAM1, MMP2, LOX, CTSK, COL6A2, TGFB1, COL6A3, COL22A1, CD44, CTSS, COL5A2, LCP1, COL6A1, LOXL2, COMP, ANXA2, THBS1, ABI3BP, COL12A1, ECM2, COL11A1, VCAN, POSTN* and *NID1*.

The top 20 upregulated and downregulated genes by PIF are tabulated with brief functional descriptions and absolute fold changes in DE (Table 1).

**Table 1:** The 40 most extreme genes by Phenotypic Impact Factor in High FE versus Low FE birds. These 40 genes have been reordered manually into functional groupings. The absolute DE expressed as a fold change has also been reported

Gene symbol	Gene name	Fold Change HFE/LFE	Gene function
*MB*	Myoglobin	4.10 Down	Iron storage, highly expressed in red, slow fibres
*MYH15*	Myosin heavy chain 15, slow	4.46 Down	Slow twitch fibre isoform
*TPM3*	Tropomyosin 3, slow	2.67 Down	Slow twitch fibre isoform
*MYOZ2*	Myozenin 2, slow	2.54 Down	Slow twitch fibre isoform
*TNNI1*	Troponin I type I, slow	3.36 Down	Slow twitch fibre isoform
*PLN*	Phospholamban	2.78 Down	Highly expressed in slow twitch fibres, substrate for cAMP-dependent protein kinase.
*COL3A1*	Collagen 3A1	2.09 Down	ECM
*COL12A1*	Collagen 12A1	2.66 Down	ECM
*FN1*	Fibronectin 1	2.01 Down	ECM
*BMP5*	Bone morphogenetic protein 5	4.23 Down	Negative regulation of IGF1 signaling
*RSFR*	Leukocyte ribonuclease A2	2.56 Down	Angiogenesis
*LY86*	Lymphocyte antigen 86	2.42 Down	Inflammation, apoptosis
*MGAT5B*	beta-1,6-N-acetylglucosaminyltransferase	3.51 Down	Synthesis of cell surface n-glycans, ECM
*NES*	Nestin	2.64 Down	Expressed in nerve cells, implies motor unit
*APOA1*	Apolipoprotein A1	2.27 Down	Promotes cholesterol efflux from tissues. Highly expressed in liver.
*MGP*	Matrix Gla protein	1.84 Down	ECM
*IGJ*	Immunoglobulin J polypeptide	2.21 Down	Little known
*SEPP1*	Selenoprotein P	2.19 Down	Extracellular glycoprotein and antioxidant
*CA3*	Carbonic anhydrase III	1.93 Down	Response to oxidative stress, expressed at high levels in skeletal muscle
*CTSS*	Cathepsin S	2.24 Down	Lysosomal cysteine proteinase
*H2AFY*	Histone family member Y	2.47 Up	Associated with lipogenic genes
*HIG2A (ENSGALG00000003348)*	Hypoxia-inducible domain family	2.15 Up	Little known
*TESK1*	Testis specific kinase	1.95 Up	Cell matrix communication
*C11ORF89* ^*^	Uannotated ORF	2.44 Up	Unannotated
*CKMT1*	Creatine kinase mitochondrial 1	1.63 Up	Creatine metabolism, muscle energy supply
*CNTFR*	Ciliary Neurotrophic factor	2.20 Up	Skeletal muscle development
*ENKD1*	Enkurin domain containing	2.19 Up	Cytoplasmic microtubule protein
*CA4*	Carbonic anhydrase 4	3.95 Up	Respiration, acid base balance, expressed on luminal surface of capillaries
*INSIG1*	Insulin induced gene 1	1.92 Up	Regulation of intracellular cholesterol concentration and steroid biosynthesis
*TTPA*	Tocopherol (alpha) binding protein	2.84 Up	Binds alpha-tocopherol a form of vitamin E, response to pH
*LINGO1*	Leucine rich repeat and Ig domain containing 1	1.72 Up	Nervous system development
*DCUN1D5*	Defective in cullin neddylation 1, domain containing 5	2.10 Up	Ubiquitin conjugating enzyme binding
*SNCG1*	Synuclein gamma	3.40 Up	Synapse protein
*C16ORF45*	Unannotated ORF	1.88 Up	Protein function unknown
*LIPG*	Endothelial lipase	2.20 Up	Lipoprotein metabolism and vascular biology
*(ENSGALG00000028983)*	BLASTS to HOXA9	2.33 Up	Transcription Factor
*(ENSGALG00000027955)*	BLASTS to MHC	2.31 Up	MHC immune function
*BFSP1*	Beaded filament structural protein 1	3.16 Up	Cytoskeletal structure
*MYL2_1 (MYL2A)*	MYL2A	3.39 Up	Thought to be more highly expressed in cardiac muscle i.e. slow fibres
*MYBPH*	Myosin binding protein H	1.53 Up	Fast isoform

^*^ We further explored the possible function of the gene encoding an unannotated Open Reading Frame (ORF) *C11ORF89*, given it was in the top 20 upregulated genes in the HFE birds. The entry in GeneCards (under the alias *PRR33*) predicts the ORF to translate into a large protein containing regions of low complexity and comparative analysis suggests that the protein was present in the ancestor of the chordates. It is located in the *G. gallus* genome adjacent to *TNNT3* and *TNNI2*, and cellular compartment analysis (http://compartments.jensenlab.org/) suggests there is moderate evidence it is a cytoskeletal protein

We also cross-referenced the genes and direction of change in Table 1 to the previous microarray study on the same samples [5]. Of the 14 genes for which we could find exact matches, 12 showed the same direction of change by the two different mRNA quantitation technologies.

### mRNA encoding the mitoproteome

Of the 699 genes encoding proteins localised to the mitochondrion, 475 were upregulated in HFE and 224 were downregulated in HFE. This is a very significant skew (binomial *P* value < 0.000001) implying the HFE birds have an elevated mitochondrial content. A similar skew was also observed in upregulated proteins in HFE breast muscle in a recent proteomics study [33]. This indicates that in general terms the upregulated genes tended to translate into upregulated mitochondrial proteins.

### Muscle model visualisation

Overlaying DE genes between HFE and LFE birds (M-values) on the VMus3D, identified a coordinated down-regulation of thick (*MYH15*, *MYL2* and *MYOM3*), thin (*TPM3*, *TNNI1*) and z-disc (*MYOZ2* and *CSRP3*) proteins encoded by slow twitch isoform genes in HFE birds (Fig. 2). Alterations of genes encoding Myosin Heavy and Light Chain proteins (*MYH15* and *MYL2*) together with the tropomyosin (*TPM3*): troponin (*TNNI1*) are components of the key protein complex in determining biomechanical efficiency of striated muscles [34] . Furthermore, the z-disk and costamere have been documented as key complexes involved in sensing mechanical stress and converting this to chemical signals [28, 35, 36]. Although no costameric proteins were identified as DE using VMus3D, the z-disk protein *CSRP3* has been associated with both structural and signal transduction roles in cardiac muscle [37] and *MYOZ2* has been linked to calcineurin signalling, a key protein phosphatase involved in fibre type regulation [38, 39]. Together these results indicate a difference in contractile protein composition and key contractile proteins involved in the regulation of fibre type. It is worth noting that in the previous microarray study that used pooled RNA samples from the HFE and LFE muscle tissue, CSRP3, MYOZ2, and TNNI1 were the number 1, 3, and 5 upregulated genes in the LFE phenotype. Thus, there appears to be good agreement between the microarray and RNAseq analysis data.

### Permut Matrix clustering on extreme PIF

After normalising on rows, clustering on columns correctly ascribed each bird into the correct group of origin (HFE or LFE) (Fig. 3). Interestingly, the dendrogram suggests the HFE birds have lower dispersion than the LFE birds. Functional groups contained in the 20 downregulated genes in HFE include slow twitch contractile subunits (*MYH15, TPM3, MYOZ2, TNNI1*) and associated slow twitch metabolic machinery (*MB* and *PLN*); components of the extracellular matrix (*COL3A1, COL12A1, FN1, MGAT5B* and *MGP*); negative regulation of IGF1 signalling (*BMP5*); and cholesterol metabolism (*APOA1*). The observed downregulation of *BMP5*, a negative regulator of IGF1 signalling, implies IGF1 signalling is increased in HFE birds. Functional groups contained in the 20 upregulated genes include mitochondrial metabolism (*CKMT1*); fast twitch muscle (*MYBPH*); and acid-base balance and response to pH (*CA4, TTPA*).

### Regulatory Impact Factor analysis

The RIF1 and RIF2 scores were plotted and manually explored for outlier Transcription Factors (TFs) (Fig. 4). Most of the data is centred close to 0, implying that the majority of TFs are not differentially connected and therefore perform the same function in the same manner in the two groups. However, there are a small number of TF that are highly differentially connected. The top 3 encode progesterone receptor (*PGR*) and two TFs that are components of progesterone signalling pathways (*GZF1* and *CITED2*; Fig. 4; Additional file 4).

### Quantitative PCR technical validation

The comparison between the RNAseq and qPCR data is outlined in Table 2. The direction of expression change was confirmed in all nine cases. In seven cases the qPCR detected a larger DE than the RNAseq.

**Table 2:** The correspondence between RNAseq and qPCR data for mRNA encoding slow twitch subunits, proteins associated with slow fibres and intramuscular fat content

Gene	RNAseq fold change (HFE–LFE)	qPCR fold change (HFE–LFE)
*MYH15*	−4.47	−33.37
*TPM3*	−2.66	−1.96
*MYOZ2*	−2.55	−6.40
*TNNI1*	−3.36	−4.69
*MYBPC1*	−1.43	−2.23
*PLN*	−2.77	−3.11
*FABP4*	−1.16	−4.44
*MB*	−4.08	−8.65
*CA3*	−1.93	−1.62

### Progesterone receptor immunofluorescence

**Fig. 5:**
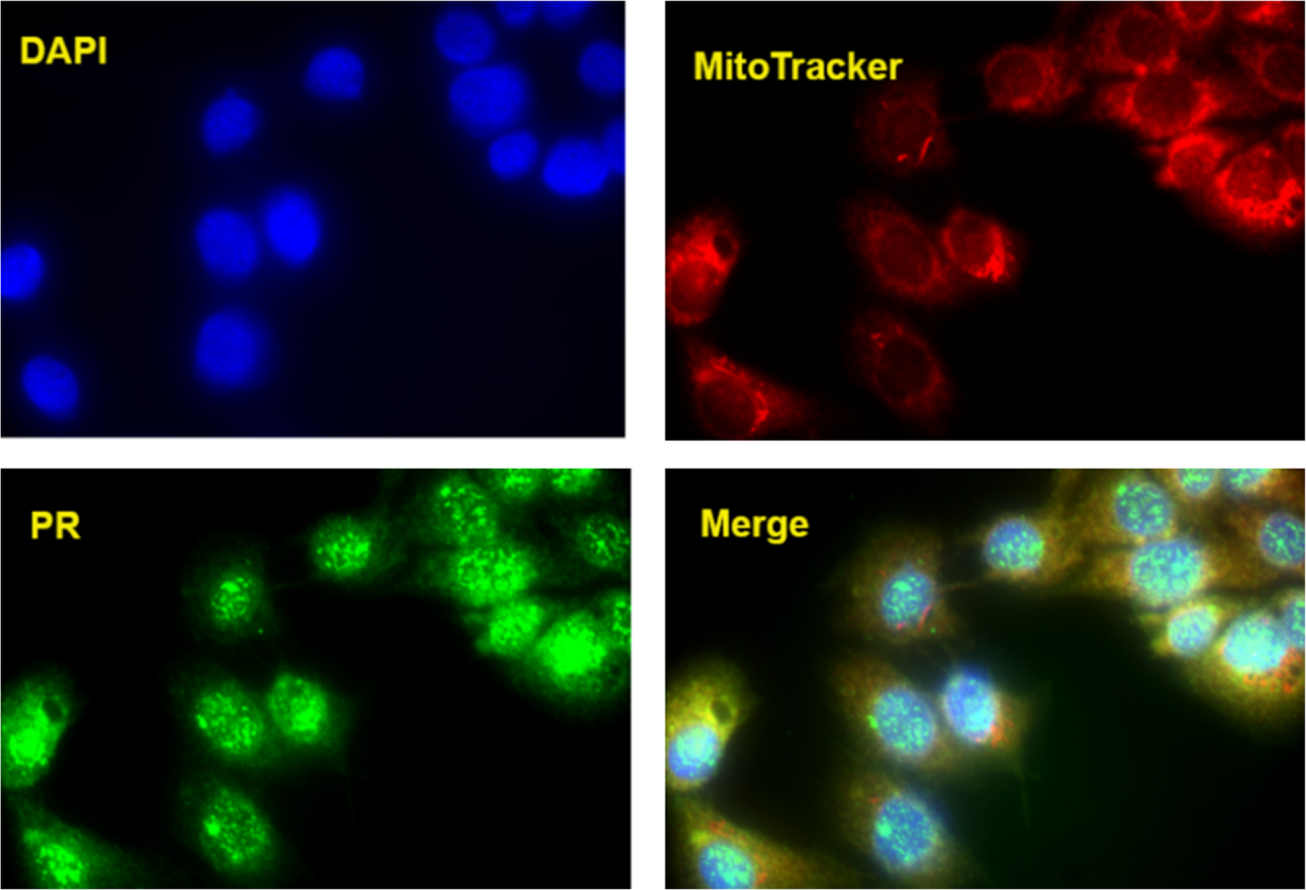
The strong co-localisation of the progesterone receptor in the mitochondria of a quail cell line

In a preliminary attempt to explore the localisation of the progesterone receptor in birds, we undertook an immunofluorescence approach in a quail muscle cell line. This qualitative approach showed a near complete overlap between the presence of mitochondria and the progesterone receptor. This indicates that most, if not all, progesterone receptor proteins are localised to the mitochondria in this particular in vitro avian muscle cell line (Fig. 5).

## Discussion

### Muscle structure

At a gross histological level, broiler pectoralis muscle is unique among the vertebrates, being almost exclusively built of fast, glycolytic type IIB fibres [40]. This muscle tissue would be expected to have a sparse capillary network, and the individual fibres to possess a low mitochondrial content and few lipid droplets. This expectation was essentially borne out in the 1970’s by [3], where a mitochondrial content of just 4% was determined by Transmission Electron Microscopy and morphometric analysis in chicken pectoralis muscle. This is approximately 10-fold less than the 35% mitochondrial content of very athletic hummingbird pectoralis muscle [41]. Collectively, these observations indicate that chickens have an unusually homogeneous breast muscle structure bearing a very low aerobic capacity. Broiler chickens are the most feed efficient vertebrates known with a dry weight in to wet weight out ratio of ~1.5 [2]. It has been previously argued that the very low aerobic capacity of their muscle is a likely driver of this phenotype through avoiding paying for ‘expensive’ spare aerobic capacity and considerations of economic physiological design [21].

Indeed, it has been observed in many independent circumstances that the most feed efficient breeds, selected on either FE or muscle mass, transition from a redder, slow, aerobic ancestral muscle to a faster, whiter, less aerobic glycolytic one in the derived state. Examples include *MSTN* mutant Piedmontese [42], Belgium Blue [43] and Blond d’Aquitaine [44] cattle, Callipyge sheep [45–47] and both Large White [48, 49] and German Landrace pigs [50]. The transition from high myoglobin content red to low myoglobin content white fibres in pigs has been so dramatic that they are no longer considered a rich source of dietary iron and are viewed as the second ‘white’ meat after chicken, rather than the third red meat after beef and lamb.

We know that these broiler muscle transcriptomes are derived (at least based on histological typing) almost exclusively from white, fast type IIB fibres [3]. In line with anatomical and physiological expectation the two most highly expressed genes in the muscle are the fast myosin heavy chain isoform *MYH1E* and the regulator of anaerobic glycolysis *GAPDH*. However, we also detected gene expression of a number of slow contractile subunits (*MYH15, TPM3, MYOZ2, TNNI1, MYOM3, CSRP3, TNNT2* and *MYL2B*) in addition to the expected fast ones. This somewhat surprising finding implies that a subset of remnant slow subunits make up a small but measurable proportion of the sarcomeric structure of the type IIB fibres, perhaps such that they are a mosaic at the molecular level.

The subtle but coordinated reduction of these slow twitch isoforms and associated slow machinery (myoglobin and phospholamban) in the higher FE birds is in line with the broad expectation of muscle ‘whitening’ being associated with high FE described above. It also concurs with our previous observations based on a cDNA microarray [5, 11]. The divergent pattern we observe in *CA3* and *CA4* may also be a simple consequence of fast versus slow fibre subunit abundance [51]. Intriguingly, while the majority of slow subunits were downregulated in HFE including *MYL2B*, a separate MYL2 isoform (*MYL2A*) was upregulated in HFE birds. We do not have an explanation for this apparent inconsistency. One would expect the expression of mitochondrial proteins to track the fibre composition data. However, our observations on the gene expression of mitochondrial proteins are not congruent with the reduction in the slow muscle fibre subunit and myoglobin/phospholamban data in HFE birds.

### Mitochondrial metabolism

In terms of mitochondrial content we found that the mRNA encoding the mitoproteome is modestly but highly significantly skewed towards the HFE birds. We propose that this indicates a slightly higher mitochondrial content and therefore a somewhat higher aerobic capacity. This is *prima facie* contrary to the reduction in slow fibre subunits and reduced myoglobin/phospholamban outlined above. It is also at odds with theoretical expectation [21] and mRNA/protein abundance data from numerous other species, particularly recent genome-wide screens performed on pigs divergent for RFI [52]. It also appears contradictory to previous work showing in chickens divergent for mass, the more efficient larger birds have a lower, not higher, mass-specific metabolic rate [53]. Having said this, we believe these specific samples have been correctly characterised. These mRNA data are in agreement with a mass spectrometry-based proteomic analysis carried out on a subset of the same breast muscle tissues (*n* = 4 per HFE and LFE groups), as the protein expression of the mitochondrial proteome was also somewhat elevated in the HFE phenotype [33].

Therefore, while the ‘big picture’ finding in production animals appears to be that selection on mass and FE reduces mitochondrial content and aerobic capacity, the data from this particular FE contrast between highly selected birds tends to point in the opposite direction. The underlying reason(s) for this notable disparity are the source of continued investigations. One possibility is that the commercial birds have such a low mitochondrial content that they are on the verge of expressing metabolically driven muscle pathologies. This would be consistent with the recent emergence of concerning phenotypes such as ‘white striping’ and ‘wooden breast.’ If a low mitochondrial content drives these pathologies, one might speculate that subtly increasing mitochondrial content may increase FE through impact on general health. Ultimately, the mitochondrial content finding has been approximated using gene expression. Ideally, this finding would be validated with an independent technology such as qPCR of mtDNA expressed relative to nDNA, but unfortunately we no longer have adequate sample material.

The general upregulation of mRNA encoding the mitoproteome in HFE birds is modest in absolute terms and there are many genes that are downregulated as well as upregulated. Extreme among the upregulated molecules in HFE are *TDRKH, MRPL55, LDHD, CKMT1A* encoding a gene silencer, a mitoribosome subunit, lactate dehydrogenase D and mitochondrial creatine kinase 1A. The biological implications of *TDRKH* for skeletal muscle are unknown, but *MRPL55*, *LDHD* and *CKMT1A* indicate an increase in mitochondrial protein synthesis (and therefore mitochondrial content), a change in redox status and increasing the transfer of high energy phosphate from the mitochondria to the cytosolic carrier, creatine. The downregulation of *PDK4*, *ACACB* and *LDHB* (encoding pyruvate dehydrogenase kinase 4, acetyl coA carboxylase beta and the B subunit of lactate dehydrogenase) indicate the HFE birds have a preference for burning carbohydrate instead of fat, a reduction in fatty acid uptake and combustion by mitochondria and a reduced reliance on anaerobic glycolysis. The first two point to a change in fuel combustion pattern that is consistent with a subtle fibre subunit transition towards a whiter muscle phenotype–after all, glycolytic fibres are geared to burn glycogen not fat during exercise. The reduction in the anaerobic *LDHB* in HFE is perhaps consistent with an elevated mitochondrial content, as the presence of higher aerobic metabolism may reduce the need to resort to anaerobic metabolism for ATP production.

### Intramuscular fat and extracellular matrix content

Although arguably not statistically significant *sensu stricto*, we wish to highlight the expression profiles of several genes previously implicated in intramuscular fat (IMF) content given the important role IMF plays in both meat quality and energetics. In the HFE birds there is a trend towards a small reduction in expression of two genes (*FABP4, PLIN2*) previously associated with amount and activity of intramuscular fat in cattle [54–57], sheep [54] and pigs [58, 59]. In absolute terms *PLIN2* and *FABP4* are 1.5 fold and 1.2 fold downregulated in HFE respectively. *PLIN2* is ranked in position 288 (extreme 3%) when the 10, 412 genes are ranked by PIF from negative (down in HFE) to positive (up in HFE) values. Together, the expression of these two genes implies the HFE birds possess a slightly leaner breast muscle. In cattle muscle, these two genes tend to be tightly co-expressed with *THRSP*, *S100G*, *CIDEA* and *CIDEC* [60], but we did not detect expression for these additional genes in the broiler data. The tendency towards higher muscle tissue leanness in the high FE birds is unsurprising, relating to the selection for overall animal mass in the calculation of Residual Feed Intake. Using basic animal mass as the selection phenotype will inevitably select against light, anhydrous but energy-dense adipose tissue and promote heavy, watery lean muscle tissue. Further, very lean meat is texturally dry and relatively flavourless–a universal problem in production animals or breeds with relatively high FE such as Callipyge sheep [61], myostatin mutant sheep [62] and Large White pigs [63].

Additionally, a very strongly coordinated reduction in expression of 41 genes encoding extracellular matrix proteins in HFE birds is consistent with less ‘white striping’, a meat defect that has recently appeared in poultry, particularly large birds grown too quickly [64]. White striping comprises seams of collagenous material containing some embedded adipocytes. The coordinate down regulation in HFE birds of genes encoding ECM components in addition to those relating to lipid content can also be seen as consistent with a reduction in this pathology.

### Progesterone signalling and FE

We have characterised the anatomical and physiological differences between high and low FE broiler muscle. The HFE bird muscle exhibits a reduction in slow muscle subunits and associated machinery and a subtly elevated mitochondrial content. But what is the ultimate cause of this muscle phenotype shift? The differential network RIF analysis clearly points to alterations in progesterone signalling as being the major driver. The 3 most differentially connected Transcription Factors are *CITED2*, *PGR* and *GZF1*. That is, the progesterone receptor itself, and two molecules that communicate progesterone signalling or are influenced by it [65, 66]. However, the RIF analysis does not imply activation or repression of the pathway, the direction of change needs to be inferred by different means. In a concurrent proteomics study being conducted on the PedM broiler line, progesterone was predicted to be activated using a commercial software program (Ingenuity Pathway Analysis, Qiagen, Valencia, CA, USA), based on expression of downstream proteins [33]. Thus, two independent analytical approaches, one based on global gene expression and the other based on global protein expression both point towards progesterone as a driver of the FE phenotype.

Progesterone is synthesised from cholesterol, so our additional identification of “cholesterol biosynthesis” (*DHCR24*, *INSIG1*, *FDPS*, *SC5D* and *MVD*) as enriched in the upregulated genes in HFE birds is noteworthy. In mammals, progesterone synthesis is known to occur in reproductive organs, adrenal glands, nervous tissue and adipose tissue. To the best of our knowledge endogenous synthesis has not been detected in mammalian skeletal muscle. The situation in birds is unknown. In fact, we could not find any published information on progesterone production nor mode of action in avian muscle.

However, in cattle exogenous administration of progesterone is often used in hormone growth promotant mixes (in concert with testosterone and estrogen) that raise FE by ~20% [67]. Indeed, the synthetic gestagen (progesterone analog), melengestrol acetate, has been found to stimulate muscle growth in yearling heifers [68]. Furthermore, in pregnant humans progesterone alters muscle phenotype from a slow oxidative to fast glycolytic state [69]. This slow to fast contractile skeletal isoform transition is certainly consistent with what we observe in our HFE broiler data (but we do not see the accompanying reduction in aerobic capacity). Progesterone also promotes protein synthesis in human skeletal muscle and rat cardiac muscle [70, 71]. Finally, a truncated isoform of the progesterone receptor localises to the mitochondrion in mammals [72]. Here, we present a first line of evidence that the progesterone receptor also localises to the mitochondria in birds, based on immunofluorescence in a quail muscle cell line. Consequently, the posited role of progesterone signalling via the progesterone receptor in driving variation in FE is a further line of evidence consistent with the mitochondrion being a central player.

### Previous work on muscle gene expression and poultry feed efficiency

We have previously documented gene expression patterns in these same samples using microarray technology [5], finding a similar reduction in the HFE birds of genes encoding a set of slow twitch contractile subunits (*CSRP3, MYH6, LMOD2, MYBPC1, TNNI1, TNNT2*) plus slow machinery *PLN* and *ATP2A2*. Another group [73] examined 23 breast muscle samples from a different population of commercial broilers also using RNA sequencing technology. Surprisingly, some of their basic structural findings are exactly opposite to ours, in that they found the slow contractile subunits (*CSRP3, MYOZ2, MYL2, TPM3, MYH15*) and intramuscular fat content (*FABP4, PLIN2*) as upregulated in the HFE birds. The reason for this very fundamental contradiction is a mystery at this stage, but may be attributable to different genetics or some other factor. Finally, we wish to emphasise that the progesterone signalling prediction made by RIF on our mRNA data has also been independently borne out by Ingenuity Pathway Analysis ‘upstream analysis’ on proteomics data derived from the same muscle samples [33]. Thus, analyses performed at two levels of biological organisation (mRNA and protein) and two independent inference strategies (RIF and IPA) have come to the same conclusion.

## Conclusions

Selection on FE at the whole bird level culminates in measurable changes in breast muscle phenotype. Despite the subtlety of the changes, we have identified a panel of 40 genes whose expression level can discriminate the two groups and whose functions include slow muscle structure (and associated machinery such as myoglobin and phospholamban) and mitochondrial activity. Hierarchical clustering shows the HFE birds show less dispersal than the LFE, reflecting a reduction in gene expression variation. This concept of reduced variation (in the treated versus control circumstance) has been observed before as a real signal of pathway constraint [74], and therefore can be taken as informative in the same way as measures of differential expression and differential connectivity. The gene expression changes in the HFE birds suggest a whiter, leaner tissue which presumably reflects the well established relationship between elevating FE on the one hand but increasing the likelihood of deficits in meat quality on the other. This exact same issue has been observed in many other highly selected production animals such as large white pigs and callipyge sheep. The differential network analysis clearly points to modifications in progesterone signalling as a key driver of the difference between the two groups. The next challenge is to harness this finding, perhaps through diet or genetics, to help drive broiler FE even higher without exogenous administration of progesterone. Any attempt to increase FE will need to be cogniscent of possible negative effects on associated meat quality traits or the health of the birds.

## Additional files

Additional file 1: PCA analysis of (A) the raw RPM read count data, (B) the log2 transformed data and (C) the quantile normalised log2 data. Green denote HFE, red denote LFE and blue are Barred Rock outgroup animals. The Barred Rock birds were used for collective normalisation but not formally analysed in this study. The outlier red sample is LFE bird 131. (TIF 96 kb)
Additional file 2: The qPCR primers used to validate the DE of a subset of genes prioritised by RNAseq. (DOCX 12 kb)
Additional file 3: The very strong relationship between RPM and RPKM in our data, using PIF as the metric for comparison. The PIF based on RPM has a correlation of 0.93 to the PIF based on RPKM. (TIF 2058 kb)
Additional file 4: The RIF1 and RIF2 output listed in full for the 898 Transcription Factors. (XLSX 69 kb)

## Abbreviations

FE: Feed efficiency
HFE: High feed efficiency
LFE: Low feed efficiency
PedM: Pedigree broiler males
