# Supplementary figures and images for: Progesterone signalling in broiler skeletal muscle is associated with divergent feed efficiency
Source: BMC Syst Biol. 2017 Feb 24;11:29. doi: 10.1186/s12918-017-0396-2 (PMC5324283; doi:10.1186/s12918-017-0396-2)

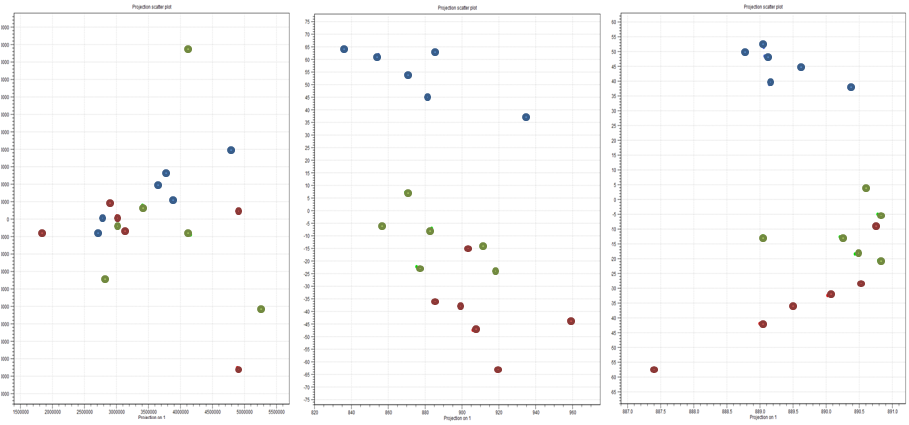

Supplement: Additional file 1: — PCA analysis of (A) the raw RPM read count data, (B) the log2 transformed data and (C) the quantile normalised log2 data. Green denote HFE, red denote LFE and blue are Barred Rock outgroup animals. The Barred Rock birds were used for collective normalisation but not formally analysed in this study. The outlier red sample is LFE bird 131. (TIF 96 kb) [file 12918_2017_396_MOESM1_ESM.tif]
