# Supplementary material for: Progesterone signalling in broiler skeletal muscle is associated with divergent feed efficiency
Source: BMC Syst Biol. 2017 Feb 24;11:29. doi: 10.1186/s12918-017-0396-2 (PMC5324283; doi:10.1186/s12918-017-0396-2)
Supplement: Additional file 2: — The qPCR primers used to validate the DE of a subset of genes prioritised by RNAseq. (DOCX 12 kb) [file 12918_2017_396_MOESM2_ESM.docx]

Additional file 2. The qPCR primers used to technically validate a subset of genes detected as DE by RNAseq.

| **Gene** | **Forward primer** | **Reverse primer** |
| --- | --- | --- |
| MYH15 | CTGGCAAGACTGTCAACACAA | CCAGTTTTGGTAGCAGGTTGA |
| TPM3 | GCATCCAGCTGGTAGAGGAG | TTTCATGCCTCTTTCGCTTT |
| MYOZ2 | GAAATCGTGGAGAGCCCATA | TCCGGGGATCAGGTGTATTA |
| TNNI1 | AGCCAACTCCAGGATCTGTG | CTCCCTGGTGTTATGGTTGC |
| MYBPC1 | ATGACAACAGGAGGCCAATC | TTCAGGTGGATTTCCTGTCC |
| MB | ACCAGGAGTGGCAACAAGTC | AAGTCTCAGGGTGGTCATGG |
| CA3 | GAGACAAACAGTCGCCCATT | CCGTTGTTCAGGATGGTTTT |
| PLN | TTTGCCTCTTGCTGATCTGT | TCTTCCTTTTGGGAGGCTTT |
| FABP4 | GAAGTGGGATGGCAAAGAGA | GCTTCCTCATGCTCTTTCGT |
